# Implicit and Explicit Memory in Youths with High-Functioning Autism Spectrum Disorder: A Case-Control Study

**DOI:** 10.3390/jcm10184283

**Published:** 2021-09-21

**Authors:** Elisa Fucà, Giulia Lazzaro, Floriana Costanzo, Silvia Di Vara, Deny Menghini, Stefano Vicari

**Affiliations:** 1 Child and Adolescent Neuropsychiatry Unit, Department of Neuroscience, Bambino Gesù Children’s Hospital, IRCCS, 00146 Rome, Italy; elisa.fuca@opbg.net (E.F.); giulia.lazzaro@opbg.net (G.L.); floriana.costanzo@opbg.net (F.C.); silvia.divara@opbg.net (S.D.V.); deny.menghini@opbg.net (D.M.); 2 Department of Human Science, LUMSA University, 00193 Rome, Italy; 3 Department of Life Science and Public Health, Università Cattolica del Sacro Cuore, 00168 Rome, Italy

**Keywords:** Serial Reaction Time Task, neurodevelopmental disorders, developmental age

## Abstract

Individuals with autism spectrum disorder (ASD) usually manifest heterogeneous impairments in their higher cognitive functions, including their implicit memory (IM) and explicit memory (EM). However, the findings on IM and EM in youths with ASD remain debated. The aim of this study was to clarify such conflicting results by examining IM and EM using two comparable versions of the Serial Reaction Time Task (SRTT) in the same group of children and adolescents with ASD. Twenty-five youths with high-functioning ASD and 29 age-matched and IQ-matched typically developing youths undertook both tasks. The ability to implicitly learn the temporal sequence of events across the blocks in the SRTT was intact in the youths with ASD. When they were tested for EM, the participants with ASD did not experience a significant reduction in their reaction times during the blocks with the previously learned sequence, suggesting an impairment in EM. Moreover, the participants with ASD were less accurate and made more omissions than the controls in the EM task. The implications of these findings for the establishment of tailored educational programs for children with high-functioning ASD are discussed.

## 1. Introduction

Autism spectrum disorder (ASD) is a complex neurodevelopmental disorder that is characterized by persistent deficits in social communication and interaction, associated with restrictive, repetitive, and stereotyped patterns of behavior, activities and interests [1]. In the US, the latest estimate of the prevalence of ASD is 1:54 [2], and the first population-based prevalence study on ASD to be conducted in Italy reported a rate of ASD of 1:87 in children aged 7–9 years [3]. 

ASD has heterogeneous manifestations because the features and severity of its symptoms can vary significantly between patients, and because its core symptoms are frequently associated with co-occurring neurological or psychiatric conditions, such as epilepsy, intellectual disability, anxiety disorders, attention deficit/hyperactivity disorder, and depression [4,5].

Atypical sensory experiences occur in up to 90% of individuals with ASD and affect all sensory modalities, predicting higher-order deficits in social and cognitive function and accounting for independent variances in social and communication symptoms [6]. In particular, perceptual representation in ASD is characterized by a relative bias toward the local over global features of a sensory scene [6,7]. This finding has led to the proposal of an “Enhanced Perceptual Functioning” model, highlighting increased perceptual expertise as a distinct feature of cognition in ASD [8]. 

Beyond the well-described abnormalities in basic sensory processes, sparse abnormalities have been reported in higher cognitive functions, such as complex information processing, executive function, memory, and motor learning, corroborating the phenotypic variability in ASD [9,10,11,12,13]. For instance, recent meta-analyses on ASD reported deficits across various domains of executive function—in particular, working memory, flexibility, and planning [14,15]—supporting the hypothesis that executive dysfunction contributes to the behavioral characteristics of ASD. 

Memory and learning in youths with ASD are crucial research topics, because abnormal learning abilities can significantly affect a person’s development. Memory impairments that arise during development can result in considerable difficulties in what and how learning takes place across the entire lifespan [16]. Thus, the characterization of specific impairments in the memory domain, such as implicit (IM) and explicit memory (EM), in children with ASD has garnered interest from clinicians and professionals, because it can be used to optimize strategies for early interventions and educational programs [9,17]. 

IM is defined as the acquisition of knowledge that occurs without the person’s awareness [18,19,20], and it is crucial for the development of fundamental skills, such as language [21,22], the ability to solve complex real-life problems [23], communication, and social skills [24,25]. IM deficits have been described in genetic diseases, including Duchenne muscular dystrophy [26] and Williams syndrome [27], and neurodevelopmental disorders, such as developmental coordination disorder [28], dyslexia [29,30,31] and language disorder [32,33]. 

EM occurs when a person intentionally acquires and retrieves information. It differs from IM because it allows the individual to develop accessible declarative knowledge that can be used deliberately during performance [34,35]. Several studies have demonstrated deficits in EM in children with intellectual disabilities [36,37] and neurodevelopmental disorders, such as language disorder [38] and attention deficit/hyperactivity disorder [39]. 

Deficits in IM have been proposed to contribute to the development of the social communication difficulties that are usually observed in ASD. For example, Mostofsky and colleagues [40] reported impaired IM in 11 children with ASD, compared with 17 controls who were matched for age and IQ, on a visual motor procedural learning task. Similarly, Gaigg and Bowler [41] found impaired fear conditioning in adults with Asperger syndrome. Subsequent studies have reported altered IM in ASD [42,43,44]. However, several studies and meta-analyses failed to confirm IM deficits in the ASD population [33,45,46,47,48,49,50].

Studies on EM in high-functioning individuals with ASD have noted largely intact EM, as tested by recognition and cued recall tests [9,51]. Consistent with these findings, individuals with ASD have been suspected of tending to resort to EM strategies in situations that are usually solved implicitly by typically developing individuals [52,53]. Similarly, the visuo-motor sequence learning in children with ASD has been found to be accurate [54] despite their tendency to repeat errors and take longer to complete the sequence. However, other research has documented the presence of specific deficits in EM subdomains. Specifically, recollection in ASD is more impaired than semantic memory and familiarity-based recognition memory, e.g., [55,56,57]. Recollection is the long-term memory process for retrieving and re-experiencing the specific details and spatial-temporal context of previous events [51,58]. In addition, deficits in autobiographical recollection have been seen in individuals with ASD due to a significant decrease in the recall of event-specific autobiographical details [59,60,61]. 

One of the major limits of the existing studies on individuals with ASD is that IM and EM are rarely assessed in the same group of participants with comparable tasks. Only one study has compared children with ASD and controls with regard to IM and EM, using the same experimental paradigm in two groups with ASD, reporting a deficit in EM but not IM [47]. 

The aim of this study was to examine IM and EM using two comparable versions of the Serial Reaction Time Task (SRTT) [62] in the same group of youths with high-functioning ASD. Consistent with meta-analyses that have observed the relative preservation of IM in ASD, e.g., [46], we predicted that our group of children and adolescents with ASD would be more impaired in EM than IM. 

## 2. Materials and Methods

### 2.1. Participants

Youths with ASD were recruited from the Child and Adolescent Neuropsychiatry Unit of Bambino Gesù Children’s Hospital (Rome). All of the patients underwent an extensive examination by a team of neuropsychiatrists and psychologists with specific expertise in assessing ASD. The diagnosis of ASD was established per the *Diagnostic and Statistical Manual of Mental Disorders,* 5th Edition [1], and was based on an accurate reconstruction of the clinical history of the patient, direct observation, and the Autism Diagnostic Observation Schedule, 2nd Edition [63]. Neurosensory deficits and comorbid psychiatric and neurodevelopmental conditions were excluded, based on the developmental history and the extensive clinical examination.

Non-verbal Intelligence Quotient (nvIQ) was assessed with the Global Non-Verbal Intelligent Quotient of the Leiter-3 scale [64], or the Perceptual Reasoning Index of the Wechsler Intelligence Scale for Children Fourth Edition [65]. The inclusion criteria comprised the following: (i) age, 6–17 years; (ii) absence of intellectual disability (IQ > 70); and (iii) absence of drug-resistant epilepsy, attention deficit/hyperactivity disorder, and neurosensory deficits. 

The typically developing participants (TD) without a history of psychiatric disorders were recruited from local primary and secondary schools. They underwent the same IQ evaluation. Although the data on the standardized instruments for screening and diagnosing ASD were unavailable for the TD, the parents and teachers confirmed that none had any special education needs, a history of ASD, or other documented behavioral disorders.

Prior to the experiment, all of the participants and their parents gave informed consent per the Code of Ethics of the World Medical Association (Declaration of Helsinki), and the study was approved by the Bambino Gesù Children’s Hospital Ethical Committee (Protocol Number 486LB).

### 2.2. Procedures

All of the participants were tested individually in a quiet, well-lit room. The participants received specific instructions for the execution of the experimental tasks, and before starting the test phase, they were asked to repeat the instructions to confirm their comprehension. The total duration of each experiment was approximately 16 minutes. IM and EM were evaluated using two versions of the SRTT, as reported [66]. 

In brief, both versions of the SRTT consisted of a reaction-timed keypress response, following visual cues. The tasks started with the participant staring at 4 horizontally arranged empty boxes that were projected onto a computer screen. Each box corresponded to 1 of 4 keys on the keyboard. The participant was instructed to put his/her left middle and index fingers on the C and V keys of the keyboard, and his/her right index and middle fingers on the B and N keys, respectively. At fixed intertrial intervals of 1667 milliseconds (ms), one of the boxes turned red, and the participants had to press the corresponding key as quickly and accurately as possible. As soon as the subject pressed a key (regardless of accuracy), the box returned to the baseline color (colorless). Accordingly, the interval between 2 red boxes varied with the reaction time; thus, any anticipation of the stimulus could be prevented.

The boxes turned red in a pseudorandom order, with the constraint that the same box could not be highlighted in 2 subsequent trials. The 2 experiments were administered on 2 consecutive days; Experiment 1 was always performed on the first day, in order to avoid carry-on effects.

#### 2.2.1. Implicit Memory Serial Reaction Time Task

The stimuli were presented in 6 blocks, each consisting of 54 stimuli. During Blocks I (R1imp) and VI (R6imp), the stimuli were presented in a pseudorandom order, with the constraint that the same box could not be highlighted in 2 subsequent trials. In the remaining blocks (F2imp, F3imp, F4imp, and F5imp), the stimuli were presented in a 9-element repeated sequence (corresponding to the following keys on the keyboard: BVNCVBCNV) that recurred 6 times, for a total of 54 stimuli. The median reaction times (RTs), errors, and omissions for each participant in each block were calculated. A decrease in RT was expected during Blocks F2imp—F5imp, in which the 9-item sequence was repeated. The presence of IM was verified by analyzing the changes in RT between the last 2 blocks: F5imp and R6imp (RTs should be higher in R6imp). 

The participants were not informed of the repetition of the 9-element sequence, and at the end of the task, they were asked whether the red box was patterned. Next, in order to determine whether they had gained declarative knowledge of the repeated sequence, they were informed of the presence of a specific sequence and were asked to reproduce the sequence on the keyboard by making a series of 12 keystrokes. 

The degree of declarative knowledge that was gained was measured by calculating the percentage of the items in the ordered sequence that was correctly reproduced. The longest consecutive string of correct responses was compared with a chance (binomial) distribution. At most, 4 consecutive elements were correctly assessed; however, an individual score of 5 or more elements was needed to confirm significant explicit knowledge. Thus, the individual scores confirmed a performance that was below the average guess rate, indicating the absence of declarative knowledge of the sequence. Twenty-five youths with ASD completed Experiment 1, and the measures that were collected were included in the analysis. 

#### 2.2.2. Explicit Memory Serial Reaction Time Task

In order to examine the presence of EM, the participants were first asked to memorize a 9-item sequence (corresponding to the following keys on the keyboard: BCNBVNCVC) of stimuli and repeat it as many times as necessary until they had learned it. The test began only when the participant correctly repeated the entire sequence verbally and correctly reproduced the sequence on the keyboard by making 9 keystrokes. 

As in the IM SRTT, the stimuli were presented in 6 blocks, each consisting of 54 stimuli. during Blocks I—III (F1exp–F3exp), the stimuli presentation followed the previously learned sequence that recurred 6 times, for a total of 54 stimuli in each block. In Blocks I—III, the participants were aware of the repeated pattern of the stimuli. In Block IV (R4exp), the stimuli were presented randomly without the subjects being notified. In Block V (R5exp), the stimuli were presented randomly, but the participants were informed of this scrambling. Finally, in Block VI (F6exp), the learned sequence was presented again without the subjects being notified. 

The median RTs, errors, and omissions for each participant in each block were calculated. An increase in RT was expected during Blocks R4epx and R5exp. The presence of explicit learning was verified by analyzing the changes in RT between F3exp and R4exp (RTs should be higher in R4exp), and between R5exp and F6exp (RTs should be lower in F6exp).

Eighteen of the 25 youths with ASD completed Experiment 2, and the measures were included in the analysis.

### 2.3. Statistical Analyses

The median RTs of Experiments 1 and 2 were analyzed by means of multivariate analysis of variance (MANOVA), with Group as the between-subject factor, and Block (from I to VI) as the within-subject factor. The rates of errors and omissions were calculated separately for random and fixed blocks in Experiments 1 and 2. The rates of errors in the random blocks were calculated by considering the percentage of the number of errors in the 2 random blocks of Experiments 1 and 2 of the total number of trials [Experiment 1: (number of errors in R1imp and R6imp × 100)/108; Experiment 2: (number of errors in R4exp and R5exp × 100)/108]. The same procedure was applied for the number of omissions (Experiment 1: (number of omissions in R1imp and R6imp × 100)/108; Experiment 2: (number of omissions in R4exp and R5exp × 100)/108]. 

Similarly, the rate of errors and omissions in the 4 fixed blocks was calculated [Experiment 1: (number of errors in F2imp, F3imp, F4imp, and F5imp × 100)/216; Experiment 2: (number of errors in F1exp, F2exp, F3exp, and F6exp × 100)/216). The same procedure was applied for the number of omissions [Experiment 1: (number of omissions in F2imp, F3imp, F4imp, and F5imp × 100)/216; Experiment 2: (number of omissions in F1exp, F2exp, F3exp, and F6exp × 100)/216]. 

The rate of errors and omissions of Experiments 1 and 2 were analyzed by MANOVA, with Group as the between-subject factor and Block (Random, Fixed) as the within-subject factor. Post-hoc analyses (Tukey’s HSD test) were performed. Partial eta squared (η*p*^2^) was used as an effect size, and the significance level was set at *p* < 0.05.

## 3. Results

### 3.1. Demographics

Twenty-five children and adolescents with high-functioning ASD (mean age: 10.31 ± 3.09 years; mean nvIQ: 101.60 ± 16.34) and 29 typically developing children and adolescents (TD: mean age: 10.47 ± 2.87 years; mean nvIQ: 103 ± 7.86) were included. The two groups did not differ in age (t(52) = −0.20, *p* = 0.84] or nvIQ [t(52) = -0.41, *p* = 0.68].

### 3.2. Implicit Memory Serial Reaction Time Task

According to the MANOVA of the median RTs, there was no effect of Group [F(1, 52) = 0.13, *p* = 0.72, η*p*^2^ = 0.002], whereas a significant effect of Block [F(5, 260) = 12.82, *p* < 0.00001, η*p*^2^ = 0.20] emerged. In the post-hoc analysis on the Block effects, we noted significant differences between R1imp and F2imp, F3imp, F4imp, and F5imp (all *p* < 0.01), with higher RTs in R1imp (M ± SD = 553.02 ± 141.15 ms) compared with the other blocks with the repeated pattern of stimuli (F2imp: 506.02 ± 136.80 ms, F3imp: 481.20 ± 123.77 ms, F4imp: 491.65 ± 141.95 ms, F5imp: 476.48 ± 133.10 ms). The post-hoc analysis of the Block effect also showed a significant difference between the F3/F5imp and R6imp blocks (F3imp vs. R6imp: *p* = 0.006; F5imp vs. R6imp: *p* = 0.001), confirming the occurrence of IM in both groups. Both groups increased their RTs in the last random block (R6imp: 521.59 ± 120.95 ms) versus the the preceding F5imp. The Group × Block interaction was not significant [F(5, 260) = 1.28, *p* = 0.27, η*p*^2^ = 0.02]. 

After controlling for chronological age, the results did not change [Group, F(1, 51) = 0.08, *p* = 0.77, η*p*^2^ = 0.002; Group × Block interaction, F(5, 255) = 1.26, *p* = 0.28, η*p*^2^ = 0.02)], with the exception of the Block effect, which did not reach statistical significance [F(5, 255) = 1.73, *p* = 0.13, η*p*^2^ = 0.002].

**Figure 1:**
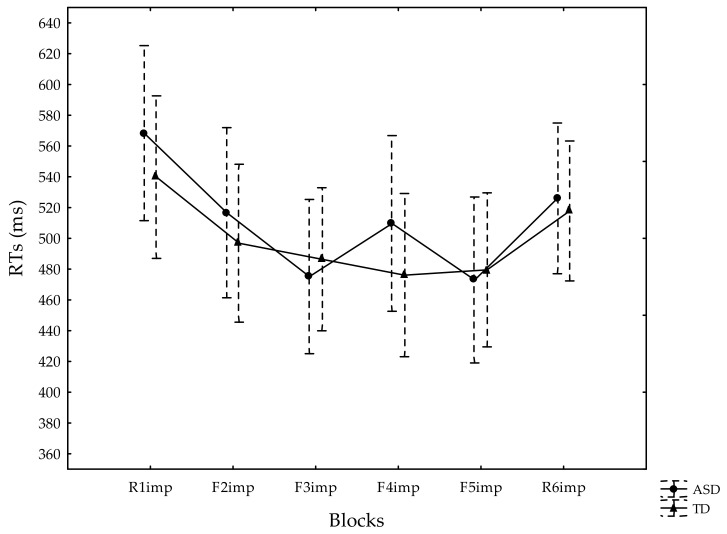
Reaction times (RTs) of participants with ASD (circles) and the TD group (triangles) in the Implicit Memory Serial Reaction Time Task. The vertical bars show the standard errors.

The median RTs of the two groups in each block are graphed in Figure 1. 

The analysis of errors found a significant effect of Group [F(1, 51) = 11.29, *p* < 0.01, η*p*^2^ = 0.18], with a higher percentage of errors in the ASD group (18.47% ± 17.98 %) versus the TD group (6.43% ± 6.89 %). The effect of Block was not significant [F(1, 51) = 0.17, *p* = 0.68, η*p*^2^ < 0.01]. A Group × Block interaction did not emerge [F(1, 51) = 0.22, *p* = 0.64, η*p*^2^ < 0.01].

After controlling for chronological age, the results did not change [Group, F(1, 50) = 11.29, *p* < 0.01, η*p*^2^ = 0.18; Block effect, F(1, 50) = 1.63, *p* = 0.21, η*p*^2^ = 0.03; Group × Block interaction, F(1, 50) = 0.23, *p* = 0.63, η*p*^2^ < 0.01].

The analysis of omissions did not reveal a significant effect of Group [F(1, 51) = 3.54, *p* = 0.07, η*p*^2^ = 0.06]. A significant effect of Block was found [F(1, 51) = 11.24, *p* < 0.01, η*p*^2^ = 0.18], documenting a higher percentage of omissions in the random blocks (6.69% ± 9.88%) than in the fixed blocks (4.84% ± 8.97%). Furthermore, a Group × Block interaction emerged [F(1, 51) = 7.80, *p* = 0.01, η*p*^2^ = 0.13]. A post-hoc analysis showed that the rate of omissions in the random blocks was higher compared to the fixed blocks in the group with ASD (10.15% ± 10.47% and 6.46% ± 7.71% respectively, *p* < 0.001) but not in TD (3.83% ± 8.52% and 3.50% ± 9.83% respectively, *p* = 0.98). 

After controlling for chronological age, the results slighty changed, with a significant Group effect [F(1, 50) = 4.28, *p* = 0.04, η*p*^2^ = 0.08], with a higher percentage of errors in the ASD group (8.30% ± 9.0%) than in the TD group (3.66% ± 9.2%). The Block effect did not reach statistical significance [F(1, 50) = 3.15, *p* = 0.08, η*p*^2^ = 0.06], whereas the Group × Block interaction persisted [F(1, 50) = 7.73, *p* < 0.01, η*p*^2^ = 0.13].

### 3.3. Explicit Memory Serial Reaction Time Task

The analysis of the median RTs showed significant effects for Group [F(1, 45) = 6.80, *p* = 0.01, η*p*^2^ = 0.13], Block [F(5, 225) = 13.96, *p* < 0.00001, η*p*^2^ = 0.24], and the Group × Block interaction [F(5, 225) = 2.62, *p* = 0.025, η*p*^2^ = 0.06]. The presence of EM was verified by analyzing the performance of both groups throughout the blocks (F1exp–F6exp). The performance changed throughout the blocks (F1exp–F6exp) in the TD group, but not for those with ASD. The post-hoc analysis of the Group × Block interaction revealed a significant increase in RT (*p* < 0.0001) between F3exp and R4exp (from 332.52 ± 146.82 ms to 448.83 ± 88.41 ms), and a significant decline in RT between R5exp and F6exp (from 445.03 ± 76.80 ms to 364.31 ± 122.26 ms) in TD, thus documenting the occurrence of EM. Conversely, in the group with ASD, the RTs between F3exp and R4exp (454.72 ± 113.84 ms and 480.94 ± 111.90 ms), and between R5exp and F6exp (499 ± 101.85 ms and 452.50 ± 93.498 ms) did not vary (*p* = 0.99 and *p* = 0.61, respectively). 

After controlling for chronological age, the results did not change [Group, F(1, 44) = 9.36, *p* = 0.004, η*p*^2^ = 0.18; Block effect, F(5, 220) = 5.45, *p* = 0.0001, η*p*^2^ =0.11; Group × Block interaction, F(5, 220) = 3.11, *p* = 0.01, η*p*^2^ = 0.07].

**Figure 2:**
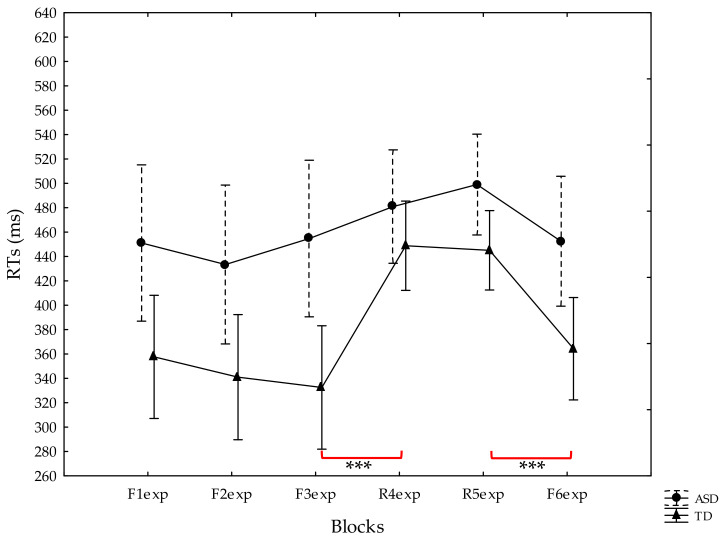
Reaction times (RTs) of the participants with ASD (circles) and TD (triangles) in the Explicit Memory Serial Reaction Time Task. Vertical bars denote standard errors. *** *p* < 0.001.

The trends in the RTs in the two groups are shown in Figure 2.

In the analysis of errors, we observed a significant effect of Group [F(1, 45) = 6.66, *p* = 0.01, η*p*^2^ = 0.13], based on the higher percentage of errors in the group with ASD (9.01% ± 8.07%) versus TD (5.12% ± 3.9%). The effect of Block was not significant [F(1, 45) = 3.37, *p* = 0.07, η*p*^2^ = 0.07]. The Group × Block interaction was not significant [F(1, 45) = 0.14, *p* = 0.71, η*p*^2^ <0.01]. After controlling for chronological age, the results did not change [Group, F(1, 44) = 7.69, *p* < 0.01, η*p*^2^ = 0.15; Block effect, F(1, 44) = 0.17, *p* = 0.68, η*p*^2^ < 0.01; Group × Block interaction, F(1, 44) = 0.17, *p* = 0.68, η*p*^2^ < 0.01].

The analysis of omissions showed a significant effect of Group [F(1, 45) = 8.11, *p* < 0.01, η*p*^2^ = 0.15], with a higher percentage of omissions in the group with ASD (3.33% ± 4.87%) than in the TD group (0.87% ± 1.42%). The effect of Block was not significant [F(1, 45) = 0.04, *p* = 0.83, η*p*^2^ < 0.001]. No significant Group × Block interaction emerged [F(1, 45) = 0.01, *p* = 0.92, η*p*^2^ < 0.001]. After controlling for chronological age, the results did not change [Group, F(1, 44) = 10.69, *p* < 0.01, η*p*^2^ = 0.20; Block effect, F(1, 44) = 0.25, *p* = 0.62, η*p*^2^ < 0.01; Group × Block interaction, F(1, 44) = 0.01, *p* = 0.91, η*p*^2^ < 0.001].

## 4. Discussion

Several studies have examined the presence of deficits in IM in ASD, generating inconsistent findings [33,42,43,44,46,47,50,67,68]. Similarly, mixed results have been reported regarding the presence of EM deficits in individuals with ASD [47,54]. In order to control for potential confounding effects due to differences in tasks, our study measured IM and EM in the same group of youths with ASD compared with a TD group, using two comparable versions of the SRTT. The main result of our study was that youths with high-functioning ASD presented with deficits on the explicit, but not implicit, version of the SRTT compared with the TD group. 

In the first experiment, youths with high-functioning ASD and TD youths experienced similar significant reductions in RT when advancing from the last block with the repeated presentation of stimuli (F5imp) to the last block with a random presentation of the stimuli (R6imp), showing similar rates of implicit learning [29,69,70]. 

Our results are consistent with meta-analyses that have reported the relative preservation of IM processes in individuals with ASD [46,48,49], based on the SRTT and other tasks, such as the alternating SRTT, Contextual Cueing [71], the Pursuit Rotor task [72,73,74], the Artificial Grammar Learning task [75,76], the Speech Stream task [77], the Observational Learning task [78], and the Probabilistic Classification task [79]. 

We can interpret our data in terms of the preserved IM in ASD, at least for certain conditions and tasks. Although it is conceivable that IM processes are adequate in individuals with ASD when they allocate their cognitive resources toward relevant stimuli in the context of structured tasks, it is possible that they experience difficulties in IM in everyday social interactions [46,80,81,82]. It has been proposed that the ability to extract regularities from continuous sensory input, which is present from the first months of life, is part of the basic cognitive skills that are required for crucial social abilities, such as understanding mental states [83,84], and that the social behavior deficits in ASD are derived from impairments in the spontaneous and rapid processing of these implicit social cues [85,86]. 

Clearly, the nature of the sequences that are to be learned in the SRTT differs in complexity and probability from those during social communication, implying an association between the elements, such as facial expression, gestures, and tone of voice [33]. Moreover, our group of participants with ASD was comprised of high-functioning youths with ASD. However, when IM has been examined in low-functioning individuals with ASD [43,44,87], deficits in IM have been documented. In particular, subsets of children with severe ASD symptoms or minimally verbal children with ASD exhibit deficits in IM compared with TD [43,44]. Notably, neuroimaging data have demonstrated a direct relationship between the severity of ASD symptoms and abnormal patterns in neural responses during an IM task [42]. Future research on IM should consider the differences between low- and high-functioning individuals with ASD to reduce the variability in the results. Moreover, it would be useful to compare the results of distinct IM tasks to determine whether the IM-spared abilities in ASD are task-dependent, and to consider the function of the other cognitive processes that are involved, such as attentional control and working memory.

In the second experiment, the TD, but not the participants with high-functioning ASD, had greater RTs from the last blocks with a repeated pattern of stimuli (F3exp) to the first random block (R4exp), and had a significant reduction in RTs when passing from the last random block (R5exp) to the last block with the learned sequence (F6exp). The absence of a significant increase in RTs during the random blocks (R4exp and R5exp) and of a significant decline in RTs during the fixed blocks suggests that individuals with ASD experience deficits in EM. Considering errors, youths with ASD made more errors than TD in both IM and EM tasks. Because SRTT requires a good level of bimanual motor coordination, our results could be linked to the motor deficits frequently reported in ASD [5]. Moreover, the higher percentage of errors exhibited by the participants with ASD could also be related to their problems with inhibition, which might cause difficulties in suppressing inappropriate responses (see below).

Our findings are consistent with those of Izadi-Najafabadi and colleagues [47], who observed that IM was preserved in children with ASD, but that their explicit learning was impaired in a motor task. Accordingly, high-functioning individuals with ASD have diminished episodic memory [88] in terms of the ability to retrieve and re-experience the specific details and spatial–temporal context of a previous event [9,58]. Although we did not specifically examine this issue, we can make some speculations on the neurobiological underpinnings of the EM deficits in ASD. A recent review on the neurocognitive bases of long-term memory in ASD summarized findings from fMRI studies [89]. Atypical lateral frontal function was found during memory encoding, perhaps reflecting the altered organization of the material that is to be learned [41,90], and during memory retrieval [91], possibly indicating a difficulty engaging top-down strategic retrieval processes. It has been suggested that atypical frontal function and frontal–posterior integration affect the top-down processing of complex information that is needed to solve EM tasks efficiently. Individuals with ASD might have less of a tendency to use relations between items to “bind” the features of an event in memory, decreasing the memory of specific “relational” contextual information that forms the basis of episodic memory [41,58,92]. A hippocampal deficit has also been proposed as a potential clarification of EM impairments in ASD. In particular, individuals with ASD have less connectivity between the hippocampus and regions of the fronto-parietal control network [91]. 

In the interpretation of our results, we should also consider executive dysfunction in ASD. Regarding the function of inhibition processes, the findings on the deficits in response inhibition in children and adolescents with ASD are mixed. Whereas some studies have reported no differences between children with ASD and TD [93,94], others have documented deficits in this domain [95,96]. Such inconsistencies could be explained by looking at three different functions of “inhibition” proposed by Friedman and Miyake [97], namely the prepotent response inhibition, the resistance to distractor interference and the resistance to proactive interference. Thus, it is conceivable that, depending on which of the three function of “inhibition” was investigated, individuals with ASD may exhibit a deficit or not. Consistent with this view, Geurts and colleagues [98] reported a weighted mean effect size of 0.55 for deficits in prepotent response inhibition, but of 0.31 for deficits in interference control in ASD. The deficit that youths with ASD showed in the EM task could be explained in part by considering that the EM task required not only the response to a current stimulus, but also the response inhibition to the following one. 

The second consideration of executive dysfunction in ASD relates to deficits in working memory. The execution of the EM version of the SRTT relies heavily on working memory for conscious storage and the manipulation of the declarative knowledge that is required to perform the task. The literature has consistently reported that individuals with ASD show deficits in verbal and spatial working memory [99]. Thus, it is conceivable that reduced working memory resources affected the EM performance in the youths with ASD. 

Collectively, our results reveal a pattern of relatively unaffected IM and diminished EM in youths with high-functioning ASD. Such a dissociation has also been observed in other conditions, such as temporal lobe epilepsy [100], Down’s syndrome [27,69], Williams syndrome [27], developmental dyslexia [29,30,31,101], and stroke-induced agrammatic aphasia [102]. 

The identification of specific abnormalities in learning processes is an important step toward establishing interventions that aim specifically to ameliorate learning disabilities in the ASD population. Our results suggest that teaching techniques that are based primarily on explicit cognitive strategies are less affordable for children and adolescents with ASD. Conversely, implicit techniques, in which rules are inferred from examples that are presented first, rather than being explicitly provided, theoretically sustain learning in children and adolescents with ASD—at least those who are high-functioning. Strategies that rely on implicit procedures are available, such as implicit social skills groups [103,104] and peer-mediated interventions [105,106]. Another approach could support EM in ASD by using specific, tailored strategies. A recent study found that the recognition ability in children with high-functioning ASD is more accurate when they can exert active control on the study experience, i.e., by choosing what and how to study [107]. However, behavioral therapeutic approaches for ASD often target explicit rules and scripts [108,109]. For instance, certain types of cognitive behavioral therapy that rely heavily on explicit knowledge improve the social skills and associated symptoms, such as anxiety, in children with ASD [110,111].

In summary, given the documented peculiarities in the learning abilities of youths with high-functioning ASD, further research into their preferred learning strategies will enhance the current teaching methods and promote novel approaches.

## 5. Conclusions

The main finding of this study was the dissociation between explicit and implicit memory abilities in youths with ASD—whereas implicit learning was preserved, explicit learning was diminished in youths with high-functioning ASD. These results have implications for tailored educational and rehabilitation programs that consider these mechanisms of learning in children with ASD. However, further studies are necessary to better characterize the learning abilities in children with high-functioning ASD and across the entire autism spectrum. 

## Data Availability

The data presented in this study are available on request from the corresponding author.
